# Correspondence

**Published:** 1877-06

**Authors:** K. E. Rich

**Affiliations:** Wenona, Ill.


### Correspondence.

WENONA, ILL., May 10, 1877.

DR. INGALS.—*Dear Sir:* I noticed in the May number of your JOURNAL a very interesting communication from Dr. E. W. Lee, "On the Best Means of Promoting Union by First Intention," by the use of fine needles for sutures.

I have been in the habit, for a good many years, of using a quite simple plan in scalp cuts, which renders shaving of the scalp unnecessary. I use neither sutures, pins nor plasters, but simply take up a small lock of hair on each side of the cut, near the margin of the wound, and tie them with the surgeon's knot. This simple procedure produces good results, and as I don't recollect of ever seeing the suggestion in any medical works, I thought it would do no harm to give the hint to the profession.

Yours truly,

K. E. RICH.

